# Assessing the variability and correlation between SUV and ADC parameters of head and neck cancers derived from simultaneous PET/MRI: A single‐center study

**DOI:** 10.1002/acm2.13928

**Published:** 2023-02-10

**Authors:** Paramest Wongsa, Mayurachat Nantasuk, Sinirun Singhnoi, Phattarasaya Pawano, Attapon Jantarato, Dheeratama Siripongsatian, Pradith Lerdsirisuk, Monchai Phonlakrai

**Affiliations:** ^1^ School of Radiological Technology Faculty of Health Science Technology HRH Princess Chulabhorn College of Medical Sciences Chulabhorn Royal Academy Bangkok Thailand; ^2^ National Cyclotron and PET Centre Chulabhorn Hospital Chulabhorn Royal Academy Bangkok Thailand

**Keywords:** head and neck cancer, intratumoral heterogeneity, simultaneous PET/MRI, SUV, and ADC

## Abstract

**Objective:**

Intratumoral heterogeneity is associated with poor outcomes in head and neck cancer (HNC) patients owing to chemoradiotherapy resistance. [^18^F]‐FDG positron emission tomography (PET) / Magnetic Resonance Imaging (MRI) provides spatial information about tumor mass, allowing intratumor heterogeneity assessment through histogram analysis. However, variability in quantitative PET/MRI parameter measurements could influence their reliability in assessing patient prognosis. Therefore, to use standardized uptake value (SUV) and apparent diffusion coefficient (ADC) parameters for assessing tumor response, this study aimed to measure SUV and ADC's variability and assess their relationship in HNC.

**Methods:**

First, ADC variability was measured in an in‐house diffusion phantom and in five healthy volunteers. The SUV variability was only measured with the NEMA phantom using a clinical imaging protocol. Furthermore, simultaneous PET/MRI data of 11 HNC patients were retrospectively collected from the National Cyclotron and PET center in Chulabhorn Hospital. Tumor contours were manually drawn from PET images by an experienced nuclear medicine radiologist before tumor volume segmentation. Next, SUV and ADC's histogram were used to extract statistic variables of ADC and SUV: mean, median, min, max, skewness, kurtosis, and 5^th^, 10^th^, 25^th^, 50^th^, 75^th^, 90^th^, and 95^th^ percentiles. Finally, the correlation between the statistic variables of ADC and SUV, as well as Metabolic Tumor volume and Total Lesion Glycolysis parameters was assessed using Pearson's correlation.

**Results:**

This pilot study showed that both parameters’ maximum coefficient of variation was 13.9% and 9.8% in the phantom and in vivo, respectively. Furthermore, we found a strong and negative correlation between SUV_max_ and ADV_med_ (*r* = −0.75, *P* = 0.01).

**Conclusion:**

The SUV and ADC obtained by simultaneous PET/MRI can be potentially used as an imaging biomarker for assessing intratumoral heterogeneity in patients with HNC. The low variability and relationship between SUV and ADC could allow multimodal prediction of tumor response in future studies.

## 1 INTRODUCTION

Head and neck cancer (HNC), including oral cavity, oropharyngeal, hypopharyngeal, and laryngeal tumors, is the seventh most common type of cancer worldwide. Recent studies have reported that the 5‐year survival rate for HNC is approximately 40–50% and accounts for 3% of cancer‐related deaths after medical treatments.^1^, ^2^, ^3^ The main effective treatment options for HNC include surgery, chemotherapy, radiation therapy, targeted therapy, and immunotherapy. The advancement of treatments improved HNC outcomes in the recent decade.^4^, ^5^ However, recurrent HNC persists and can limit curative treatments.^6^ Eckardt et al. reported that the majority of patients with HNC experienced recurrent cancer within 2 years after primary treatment.^7^

Intratumoral heterogeneity is associated with cellular and molecular characteristics such as cellular proliferation, necrosis, fibrosis, differences in blood flow and angiogenesis, cellular metabolism, tissue stiffness, hypoxia, and specific receptor expression.^8^ It poses a major challenge for the clinical management of patients with HNC owing to persistent drug tolerance within heterogeneous cell populations.^9^ This could lead to tumor recurrence after the first treatment.

Medical imaging plays a vital role in assessing intratumoral heterogeneity as it provides spatial tissue characteristics of intratumoral mass, thereby allowing heterogeneity analysis. For instance, [^18^F]‐fluorodeoxyglucose (FDG)‐positron emission tomography ([^18^F]‐FDG PET) is used to measure the therapeutic response relatively early in the course of treatment by measuring various FDG parameters, such as average standard uptake value (SUV_mean_), maximal standard uptake value (SUV_max_ or SUV_peak_), total lesion glycolysis (TLG), and metabolic tumor volume (MTV) . The TLG is the product of SUV_mean_ and MTV (TLG = SUV_mean_* MTV).

In addition to [^18^F]‐FDG PET, diffusion‐weighted magnetic resonance imaging (DWI‐MRI) is also used to investigate heterogeneity in tumor. DWI‐MRI quantification is based on the microscopic random translational motion of water molecules in biological tissues. The magnitude of translational motion is described by its apparent diffusion coefficient (ADC) values derived from DWI‐MRI images. As a result, tissues can be characterized through the variation of ADC values.^10^ Previous studies showed an inverse correlation between ADC values and cellularity in tumor, and they stated that ADC parameter can reflect tumor microstructure.^11^, ^12^ The use of ADC value was also suggested for characterizing head and neck tumors.^13^, ^14^, ^15^

Simultaneous PET/MRI is a new approach for functional and morphological imaging modality, in which [^18^F]‐FDG PET and MRI images are obtained simultaneously, allowing accurate spatially aligned multiparametric imaging to characterize intratumoral tissues.^18^ It is also a powerful tool for evaluating biology and pathology and shows great potential for assessing intratumoral heterogeneity in HNC. Meyer et al.^16^ proposed PET and MRI images’ histogram analysis, a novel technique for radiological image analysis, to assess the correlation between histogram based ADC parameter and complex FDG‐PET parameters, including SUV_max,_ SUV_mean,_ TLG_,_ and MTV in HNC patients. They found that ADC entropy had a good correlation with MTV and TLG.

Although the ADC and FDG‐based parameters have been reported that it had the potentials in clinical use in assessing tumor response, the variability in the reported accuracy of [^18^F]‐FDG PET and confounding factors, such as early reduction in activity in the presence of viable tumors or increases in uptake secondary to inflammatory processes following chemotherapy and radiotherapy, still limits the reliability of SUV parameters in assessing tumor response,^17^, ^18^, ^19^ and its clinical use for HNC prognosis is debatable. Therefore, the reliability of SUV and ADC should be investigated before adopting these parameters in clinical research because of the variability of SUV and ADC parameters obtained in a single or multi‐imaging center.^20^, ^21^, ^22^

The interscanner and intrascanner variability of quantitative parameters, in particular fractional anisotropy, of different 3T MR scanners (seven models form four vendors) obtained from nine imaging centers in 30 healthy volunteers was reported by Schlett et al. (2016).^23^ They found a higher reproducibility of intrascanner than for interscanner comparisons. The range of interscanner variability varied from 1.0% to 53.2%. They suggested that the differences decreased when using identical MRI model by single vendor. In addition, a significant difference of SUV_max_ values in phantom across 10 imaging centers was also studied by Fahey et al. (2010).^24^ They reported that SUV variability was in the range of 10–25% and suggested that the variability of SUV could be potentially increased more than this report due to biological and protocol factors.

Therefore, as the first step for assessing HNC response following the first treatment using SUV and ADC parameters as a multimodal predictor, which is derived from simultaneous PET/MRI at our center, we aimed to measure the SUV and ADC variability and also identify a correlation between their parameters in patients with HNC to ultimately develop multimodal prediction of head and neck tumor response in future study.

## 2 METHODS

As summarized in Figure 3, the study's methods were performed as follows.

### 2.1 SUV and ADC variability measurement in phantoms

In this present study, we measured variability of SUV and ADC parameters derived from PET/MRI scanner both in phantom and in‐vivo by utilizing coefficient of variation (CV) value as variability index in the rest of this work, as it is most commonly used measure for repeatability in medical imaging by many investigators.^25^, ^26^, ^27^

#### 2.1.1 SUV variability parameter measurements in [^18^F]‐FDG phantom

**FIGURE 1:**
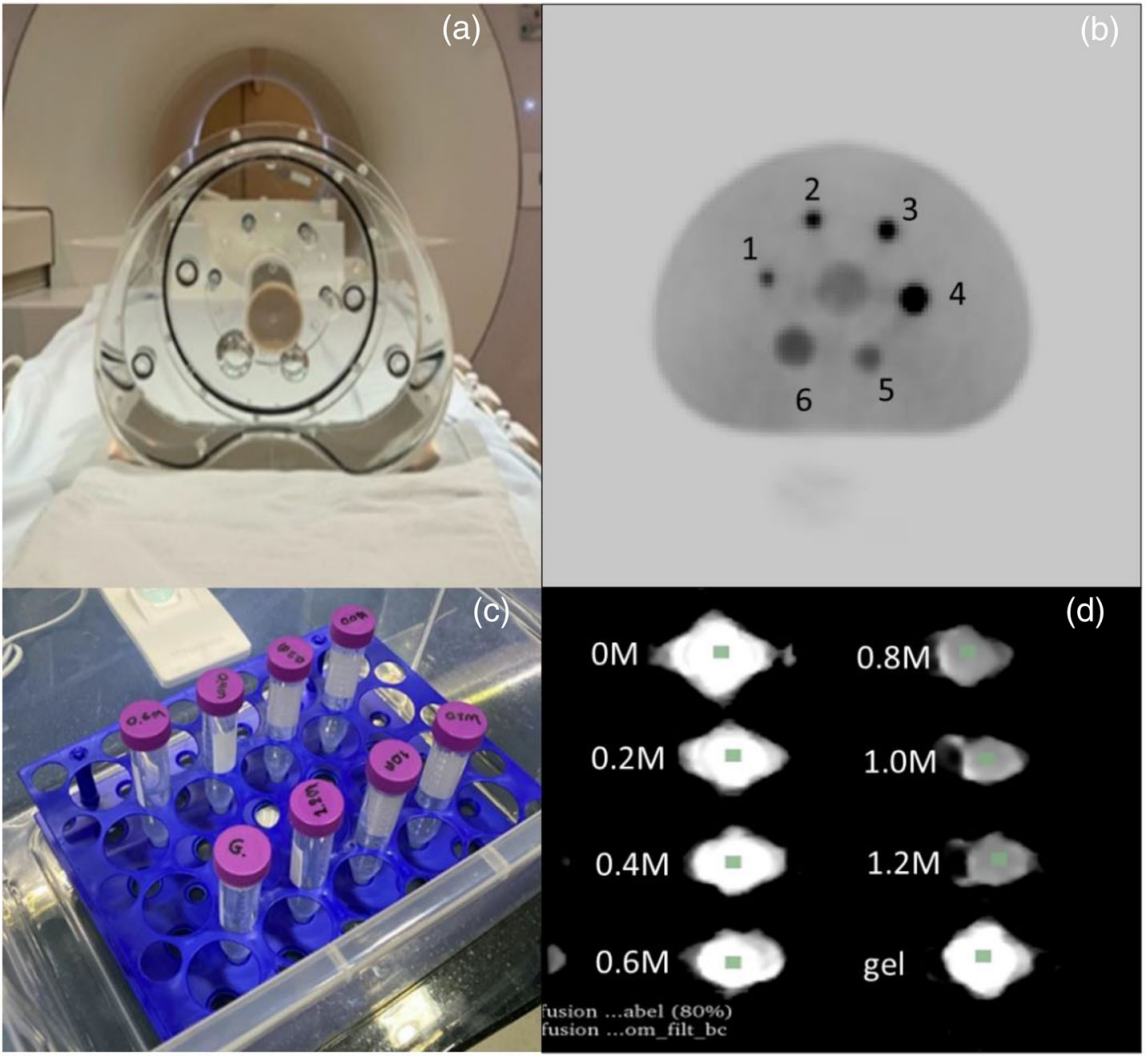
(a,b) NEMA phantom for [^18^F]‐FDG PET quality control. The phantom comprises four spheres filled with different concentrations of 259 MBq of [^18^F]‐FDG and two empty spheres (5 and 6) for reference. (c,d) In‐house diffusion MRI phantom comprising seven tubes with different concentration of sucrose solution and an ultrasound gel tube.

The SUV variability was assessed using the NEMA IEC PET Body Phantom (Model: IEC 61675–1 emission phantom), a torso‐like cavity containing six hollow spheres (internal diameters of 10, 13, 17, 22, 28, and 32 mm) surrounding a lung insert. Each sphere was filled with an [^18^F]‐FDG solution of 352.73 ± 8.46 Megabecquerel (MBq) on average at the start of the measurement, except for the spheres with diameters of 28 and 32 mm, which were empty. The phantom was placed at the center of the MRI table of PET/MRI scanner (3T Biograph mMR, Siemens Healthineer, Erlangen, Germany). Images were obtained using PET‐optimized body radiofrequency (RF) receiving coils and standard‐of‐care OSEM point spread function algorithm, as described in Table 2 for 3 consecutive days with one session per day using an identical imaging protocol used in routine simultaneous PET/MRI scans for patients with HNC, as shown in Figure 1 (a).

Three scanned datasets were imported into the 3Dslicer software (www.slicer.org) for SUV measurements. Image post‐processing was performed to remove image noise before measurements using median noise filtering with a neighborhood size of 2 × 2 × 2. Then, the region‐of‐interest (ROI) with a size of 0.19 mm^2^ was manually drawn at the center of each sphere to measure SUV. The measured SUV values across six spheres were averaged to identify the coefficient of variation (standard deviation [SD]/mean) and determine intersession variability.

#### 2.1.2 The variability of ADC parameter measurement using in‐house diffusion phantom

The ADC variability was assessed using an in‐house diffusion phantom and modified from a phantom developed by Hara et al.^28^ The phantom comprises eight tubes, each containing sucrose solution with NaN_3_ (0.03% w/w) of 0, 0.2, 0.4, 0.6, 0.8, 1.0, and 1.2 mol/L and ultrasound gel for the eight tubes as shown in Figure 1 (b).

The phantom was placed inside a PET‐optimized head/neck RF coils (16 channels). DWI images were obtained for 3 consecutive days (sessions) with three scans per session without removing the phantom. All data were acquired in the axial direction using the identical imaging protocol used in routine clinical scanning. The acquired datasets were imported into the 3Dslicer for median noise filtering (neighborhood size of 2 × 2 × 2) and N4ITK MRI bias field correction before measuring the ADC value by manually drawing a single ROI at the center of each tube in all datasets (size, 0.61 ± 0.03 mm^2^). The measured ADC values were averaged to compute both intra‐ (within session1) and intersession variability for each tube.

### 2.2 ADC variability measurement in healthy volunteers

Five normal volunteers (age, 21–22 years) were prospectively recruited for assessing the variability of ADC parameters in MRI. A signed informed consent form was obtained from all volunteers before MRI scanning. DWI‐MRI examinations of the head and neck were performed twice on the healthy volunteers with a scanning interval of 1 month using the identical imaging protocol used in patients with HNC. ADC images of all volunteers were imported into the 3Dslicer to perform N4ITK MRI bias field correction and median noise filtering (neighborhood size of 2 × 2 × 2), which computes the value of each output pixel as the statistical median of the neighborhood of values around the corresponding input pixel. This image filter method is one of efficient methods to remove “salt‐and‐pepper” noise.

The right and left parotid glands, as normal tissue references, were contoured using semi‐automatic drawing tools to measure ADC values in each patient from all datasets. ADC values were then averaged to compute intersubject variability of the parotid glands.

### 2.3 Sample size

This study was approved by the Human Research Ethics Committee of Chulabhorn Royal Academy, Thailand (Project code: 151/2564). We did not prespecify sample size and retrospectively gathered all PET/MRI data of patients with HNC who underwent simultaneous [^18^F]‐FDG PET/MRI in 2020–2021 from National Cyclotron and PET center, Chulabhorn Hospital. Radiological report was used to identify cancer types in the head and neck area. The inclusion criteria for data selection were that patients who underwent simultaneous [^18^F]‐FDG PET/MRI with a primary HNC with residual tumor mass for contouring and with motion artifact‐free images. Only 11 patients (three women and eight men; aged 48–78 years) met the inclusion criteria and were used for further analysis. The patients’ characteristics are shown in Table 1.

**TABLE 1:** Patients’ characteristics.

No.	Sex	Age (years)	MTV (cm^3^)	Diagnosed HNC
1	M	62	371.60	Right parotid gland cancer
2	M	69	1.32	Left nasopharynx cancer
3	M	53	24.11	Right submandibular gland cancer
4	M	60	1.60	Right tongue cancer
5	F	65	0.74	Right parotid and submandibular gland cancer
6	F	48	39.42	Right tongue cancer
7	M	53	4.82	Nasopharynx cancer
8	M	78	1.16	Left retromolar trigone cancer
9	M	68	35.13	Right nasopharynx cancer
10	F	59	5.47	Left nasopharynx cancer
11	M	60	278.10	Mandibular cancer

### 2.4 Simultaneous [^18^F]‐FDG PET/MRI scans

Simultaneous head and neck PET/MRI was performed using a fully integrated whole‐body PET/MRI scanner (3T Biograph mMR, Healthineer, Erlangen, Germany). Patients were asked to fast for 4–6 h, and blood sugar level (dextrostix) was measured before the examination as per routine practice. Before the examination, a dose volume (approximately 3–5 mL) of 158.10 ± 59.55 MBq of [^18^F]‐FDG was intravenously injected into the vein in the arm. Thereafter, patients were instructed not to do any strenuous movements for 60 min before the scan. This allows for minimizing the injected radiotracer uptake into the muscles or tissues because of brain activation. During the examination, patients were positioned headfirst supine to obtain static PET/MRI images simultaneously using routine head and neck imaging protocol. Single‐shot spin‐echo diffusion EPI MRI acquisition was performed using an attenuated head/neck RF coil (specific PET/MRI coils). The T1‐weighted (T_1_w) turbo spin‐echo DIXON MR sequence was utilized for attenuation correction (AC) by segmenting tissues into the four tissue classes of adipose tissues, soft tissues, lung adaptive, and bone, as well as air‐filled spaces for the synthesis of head and neck attenuation correction map (µ‐map). PET images were corrected according to the following methods: normalization, dead‐time correction, attenuation correction, scattering correction with relative model‐based, and decay correction. The parameters of image acquisition and image reconstruction were provided in Table 2.

**TABLE 2:** Simultaneous PET/MRI imaging parameters.

	Imaging Technique	
Parameter	DWI‐MRI	PET AC
Magnetic Field Strength	3T	–
Orientation	Axial	Craniocaudal
Phase direction	Anterior‐posterior direction	–
Radiotracer	–	[^18^F]‐FDG
Injection dose (MBq/kg)	–	2.59
Reconstruction method	–	OSEM+PSF (HD‐PET)
Number of iterations	–	Iterations 3 Subset 21
FOV (mm.)	250 × 173	258 × 588
Matrix size	220 × 152	256 × 256 (acquired)
Zoom factor		1
Filter		3D Gaussian
FWHM (mm.)		6.0
Scatter correction		Relative
Voxel size (mm.)	1.1 × 1.1 × 4.0	2.3 × 2.3 × 5.0
Slice thickness (mm.)	4	5
TR (ms.)	5850	–
TE (ms.)	61	–
Scan time (min)	4.45	10
b‐value (s/mm^2^)	0 and 800	–
Number of beds (PET)	N/A	1

**Abbreviations**: AC, Attenuation correction; and ms, millisecond; FOV, Field of view; FWHM, Full width at half maximum.; MBq/kg, Megabecquerel/kilogram; mm, millimeter; OSEM, Ordered subset expectation maximization; SNR, Signal to noise ratio; TE, Time of Echo; TR, Repetition time.

### 2.5 Correlation between ADC and SUV parameters in patients with HNC

**FIGURE 2:**
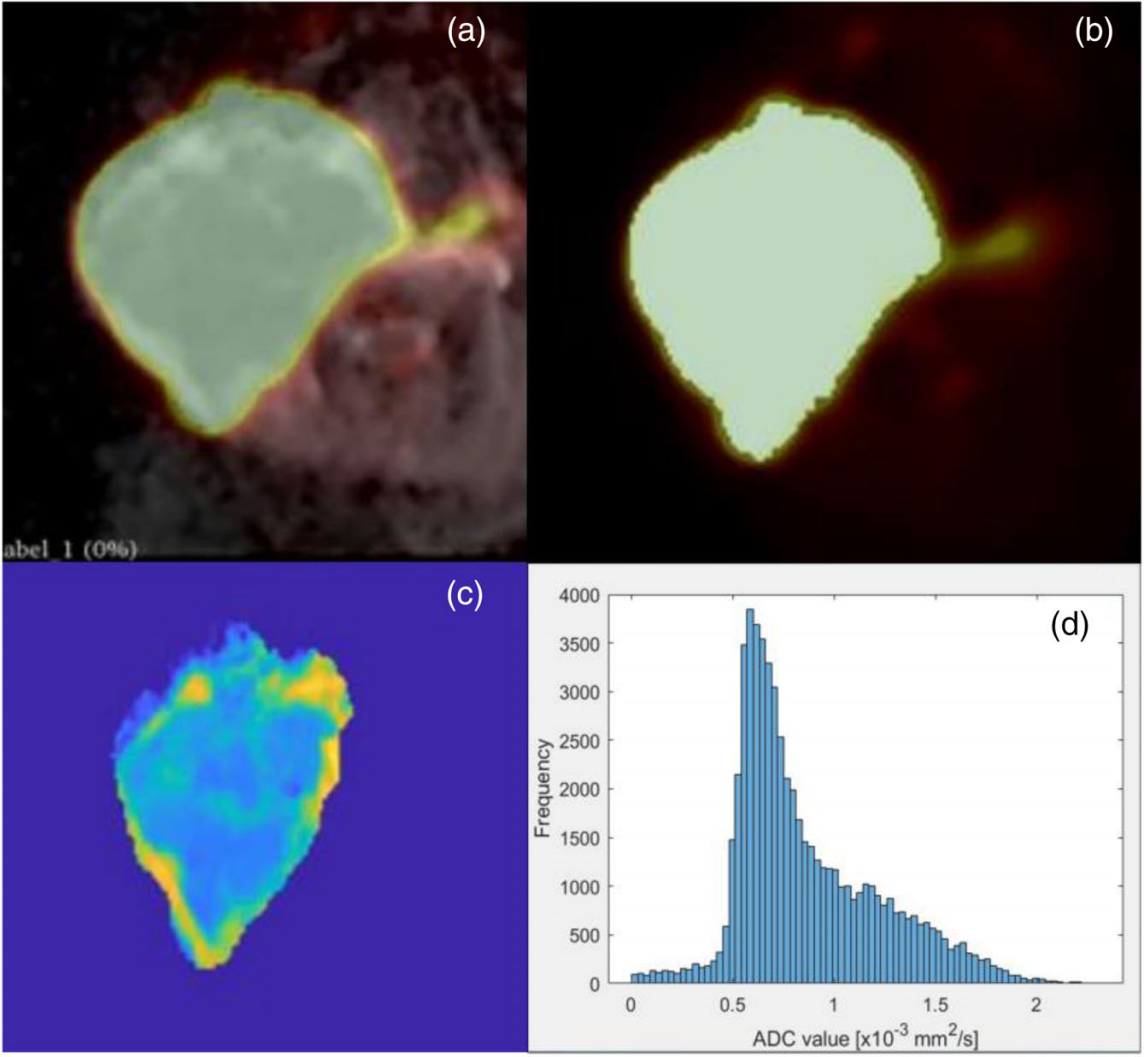
ADC image overlaid by [^18^F]‐FDG PET AC image and tumor contorting (a) and its corresponding binary mask volume (b). Segmented tumor mass (c) and its corresponding ADC histogram (d).

We utilized histogram analysis method as it was reported as an effective method for tumor heterogeneity. First, the tumor mass was contoured by a nuclear medicine radiologist using an [^18^F]‐FDG PET attenuation correction (AC) image. Then, PET AC and ADC images and tumor contours were imported into the 3Dslicer for image post‐processing, including median noise filtering (neighborhood size of 2 × 2 × 2) and N4ITK MRI bias field correction for ADC images. Tumor contouring volume was converted into a binary mask volume. Next, all post‐processed images and binary mask volume were exported as a nifty file before importing into the MATLAB software (MATLAB ver. R2022a) for image segmentation (Figure 2). Finally, the histogram of segmented tumor mass in SUV and ADC parameters was extracted to compute the ADC_mean_ and ADC_median_ values denoted as ADC_med_; ADC_max_, ADC_min,_ and ADC_Kurtosis_ denoted as ADC_kur_; ADC_Skeness_ denoted as ADC_ske_ and ADC_5,10,25,50,75,90, and 95percentiles_; SUV_mean_ and SUV_med_ denoted as SUV_med_, SUV_max_, and SUV_min_; SUV_Kurtosis_ denoted as SUV_kur_; and SUV_Skewness_ denoted as SUV_ske_ and SUV_5,10,25,50,75,90, and 95 percentiles_ variables, respectively, to assess a correlation across these extracted variables. In addition to statistic variable extraction, TLG parameter was calculated by multiplying MTV with SUV_mean_ for each patient.

**FIGURE 3:**
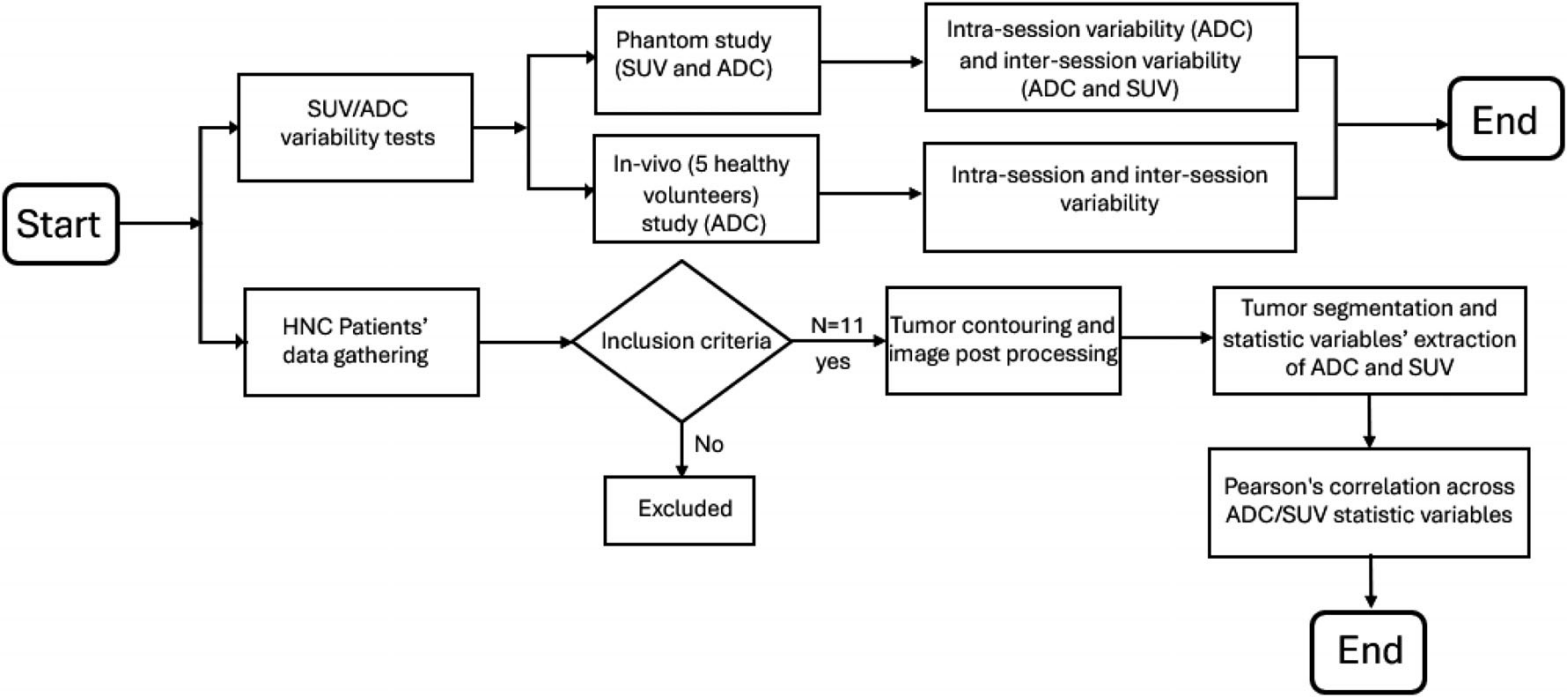
Workflow diagram of methods for assessing variability in phantom and in vivo, as well as correlation between ADC and SUV in head and neck cancer patients.

### 2.6 Statistical analyses

Microsoft Excel was used to calculate descriptive statistics. SUV values measured from the NEMA phantom were expressed as the mean and SD to compute intersession variability (SD/mean). ADC values measured from in‐house diffusion MR phantom were also expressed in the mean and SD to measure intrasession CV, whereas intersession CV of ADC was computed from the averaged ADC values and SD across sessions. For ADC variability measurements in vivo, the average ADC values and SD of the right and left parotid gland across two scanning sessions were used to measure intersubject variability.

We tested patient's data normality by Shapiro‐Wilk test using SPSS software (SPSS ver.29) to determine the normal data distribution. Pearson's correlation was used to see if there is a linear relationship between two quantitative variables between the average values of extracted ADC and SUV variables, as well as standard TLG and MTV parameters, using a MATLAB's function, *[R,P] = corrcoef()*, which provides the Pearson correlation coefficient (r) of two random variables. *P*‐values of < 0.05 were considered statistically significant. If the correlation coefficient (r) value lies near ± 1, between ± 0.5 and ± 1, between ± 0.30 and ± 049, and below ± 0.29, it is considered to show a perfect, strong, moderate, and low degree of correlation, respectively.

## 3 RESULTS

### 3.1 SUV and ADC variability measurements in phantoms

**FIGURE 4:**
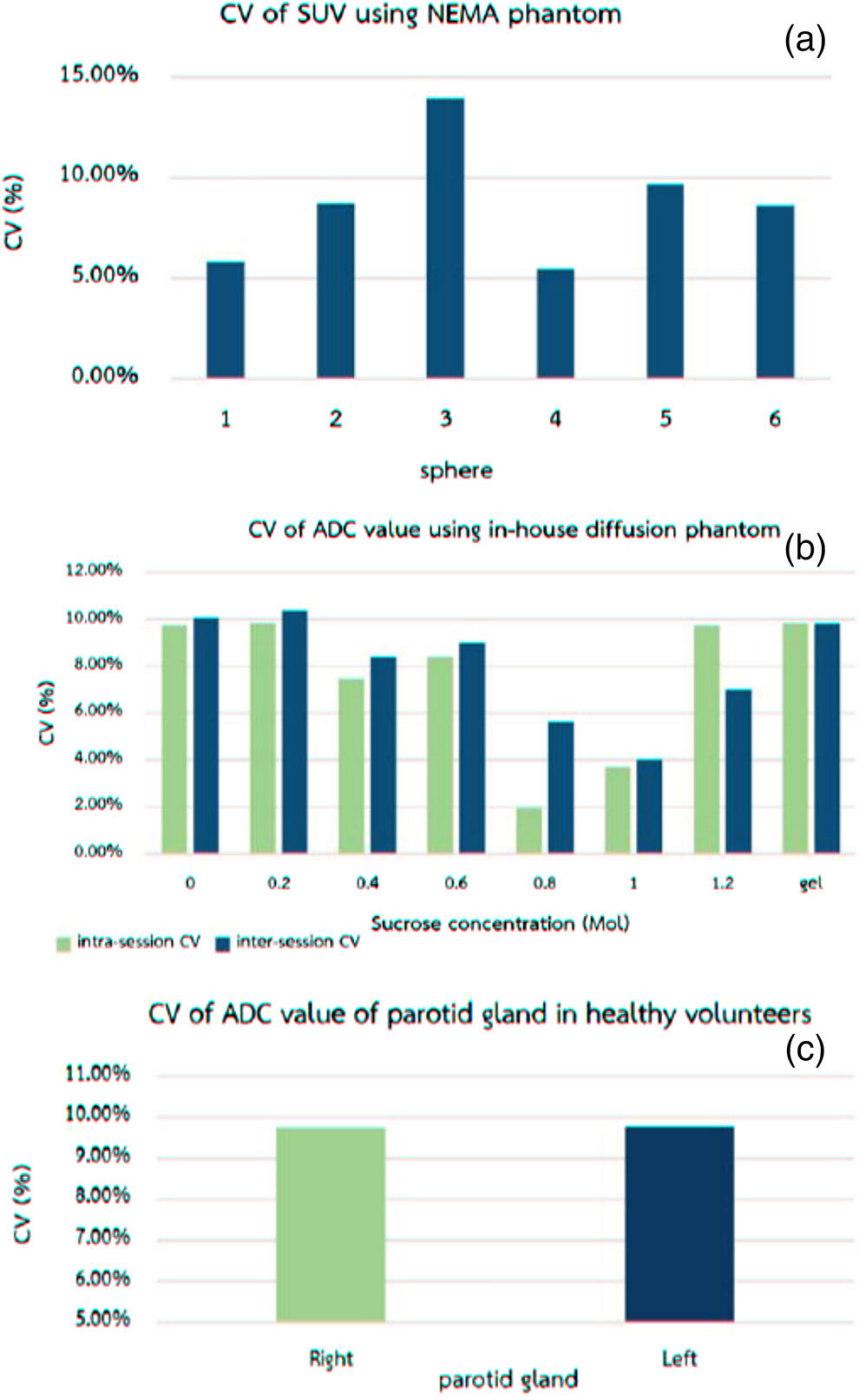
(a) Comparison of intersession SUV variability parameters in each sphere measured in NEMA phantom. (b) Comparison of ADC intrasession (in session 1) represented in green color bars and intersession variability across eight tubes in the in‐house diffusion phantom. (c) Comparison of ADC intersession variability of the right (green) and left (blue) parotid glands in healthy volunteers.

The SUV's intersession variability of the first to the eighth spheres in the NEMA phantom were 5.8%, 8.7%, 13.9%, 5.5%, 9.7%, and 8.6%. The intersession variability of all spheres was < 10%, except the third sphere (13.9%). Figure 4‐(a) shows the comparison of intersession CV across all spheres.

### 3.2 ADC variability measurement in the in‐house diffusion phantom

The ADC's intrasession variability of the first to the eighth tube was 7.9%, 8.7%, 5.8%, 4.1%, 9.3%, 7.9%, 7.9%, and 8.0%, respectively, whereas the intersession variability of each tube was 10.0%, 10.4%, 8.4%, 9.0%, 5.6%, 4.0%, 7.0%, and 9.8%.

Figure 4‐(b) shows the comparison of intra‐ and intersession variabilities of each tube containing different sucrose concentrations and ultrasound gel in the last tube.

### 3.3 ADC variability measurement in healthy volunteers

Figure (4‐c) shows that the intersubject variability of the ADC value measured in healthy volunteers was 9.7% for the right parotid gland and 9.8% for the left parotid gland. The ADC's intersubject variability of < 10% in both the parotid glands was observed.

### 3.4 Correlation between ADC and SUV parameters in patients with HNC

With the normality of 11 patient data for average SUV and ADC variables using Shapiro‐Wilk test (*P* = 0.247 and *P* = 0.181, respectively), Table 3 shows the correlation coefficient value (*r*‐value) across SUV and ADC variables. An *r‐*value is an indicator of the strength of the linear relationship between ADC and SUV variables. An *r*‐value of > 0 indicates a positive relationship; < 0 signifies a negative relationship, and 0 indicates no relationship between the ADC and SUV variables. Asterisk indicates a significant correlation (*P* < 0.05). With small data, MTV and TLG parameters had a negative correlation with ADC_mean_, ADC_med_, ADC_min_, and ADC values at 5, 10, 25, and 75 percentiles, but was not significantly correlated. ADC_mean_ and ADC_med_ had a good and significant negative correlation with SUV_mean_, SUV_med_, SUV_max_, SUV_25_, SUV_50_, SUV_75_, SUV_90_, and SUV_95_. The ADC_5_, _10, 25,_ and _50_ had a good and significant negative correlation with SUV_mean, med, max, 25,50,75, 90,_ and _95_. The ADC_75_ had a good and significant negative correlation with the SUV_mean, med, 25,_ and _50_. No significant correlation was observed between ADC_90 and 95_, ADC_ske_, and ADC_kur_ and all SUV variables

**TABLE 3:** Correlation coefficient (*r*‐value) between SUV and ADC variables.

**Variable**	ADC_mean_	ADC_med_	ADC_min_	ADC_max_	ADC_5_	ADC_10_	ADC_25_	ADC_50_	ADC_75_	ADC_90_	ADC_95_	ADC_ske_	ADC_kur_
MTV	−0.31	−0.42	−0.60	0.57	−0.52	−0.50	−0.50	−0.42	−0.16	0.10	0.19	0.06	−0.12
TLG	−0.38	−0.50	−0.52	0.45	−0.57	−0.55	−0.57	−0.50	−0.24	0.02	0.11	0.12	−0.12
SUV_mean_	−0.67*	−0.74*	−0.27	−0.15	−0.67*	−0.71*	−0.72*	−0.74*	−0.61*	−0.53	−0.43	0.22	0.13
SUV_med_	−0.68*	−0.76*	−0.30	−0.11	−0.70*	−0.73*	−0.75*	−0.76*	−0.61*	−0.51	−0.41	0.22	0.10
SUV_min_	−0.38	−0.35	0.21	−0.52	−0.24	−0.29	−0.28	−0.35	−0.42	−0.55	−0.53	0.12	0.17
SUV_max_	−0.65*	−0.75*	−0.40	0.06	−0.71*	−0.74*	−0.75*	−0.75*	−0.55	−0.41	−0.30	0.23	0.10
SUV_5_	−0.51	−0.51	−0.01	−0.40	−0.42	−0.47	−0.45	−0.51	−0.51	−0.58	−0.52	0.12	0.17
SUV_10_	−0.54	−0.55	−0.06	−0.34	−0.46	−0.51	−0.50	−0.55	−0.53	−0.57	−0.49	0.16	0.19
SUV_25_	−0.65*	−0.69*	−0.19	−0.26	−0.61*	−0.65*	−0.65*	−0.69*	−0.61*	−0.59	−0.49	0.20	0.18
SUV_50_	−0.68*	−0.76*	−0.30	−0.11	−0.70*	−0.73*	−0.75*	−0.76*	−0.61*	−0.51	−0.41	0.22	0.10
SUV_75_	−0.67*	−0.75*	−0.32	−0.07	−0.70*	−0.74*	−0.75*	−0.75*	−0.58	−0.48	−0.38	0.21	0.07
SUV_90_	−0.66*	−0.74*	−0.31	−0.07	−0.68*	−0.72*	−0.73*	−0.74*	−0.58	−0.48	−0.37	0.24	0.12
SUV_95_	−0.66*	−0.74*	−0.32	−0.05	−0.68*	−0.72*	−0.73*	−0.74*	−0.58	−0.48	−0.37	0.24	0.13
SUV_ske_	0.27	0.33	0.30	−0.08	0.28	0.31	0.36	0.33	0.20	0.07	0.00	−0.07	0.05
SUV_kur_	0.26	0.25	0.01	0.32	0.18	0.23	0.25	0.25	0.24	0.30	0.31	0.06	0.06

* Significant correlation (*P* < 0.05) using Pearson's correlation.

**FIGURE 5:**
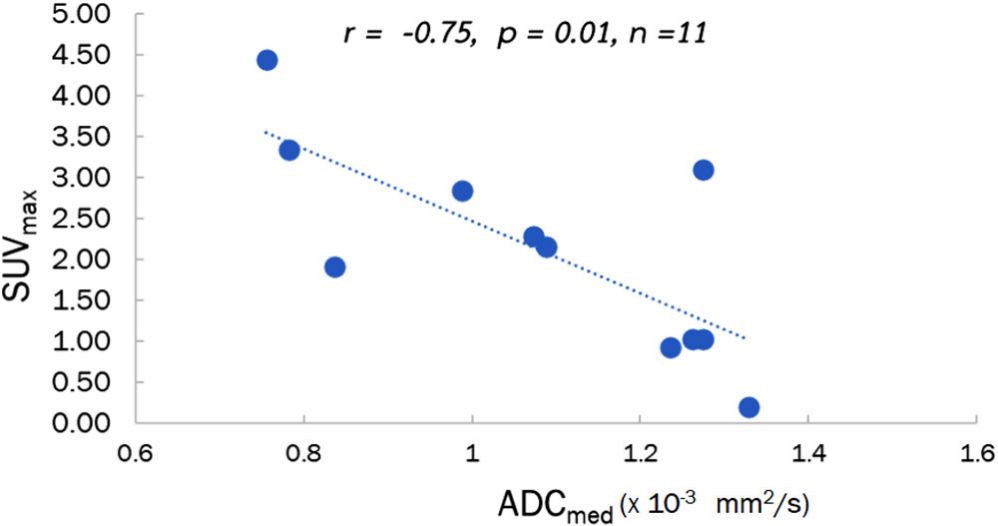
Invert linear correlation observed between ADC_med_ and SUV_max_ variables. SUV_max_ tended to increase with the decrease in ADC_med_ in HNC.

Figure 5 shows an example of a linear correlation between ADC and SUV variables (ADC_med_ and SUV_max_). The SUV_max_ value tended to increase with decrease in ADC value in patients with HNC.

## 4 DISCUSSION

This study investigated the variability of quantitative SUV and ADC parameters of a head and neck imaging protocol using simultaneous [^18^F]‐FDG PET/MRI. Our study revealed that the maximum SUV variability was up to 13.9% in the third sphere, which might be caused by motion and attenuation correction problem in the NEMA phantom. Further investigation of this issue using PET/CT for comparison is recommended to warrant the reliability use of attenuation correction method in the phantom. Furthermore, the maximum intrasession variability of ADC was up to 9.8% compared with an intersession variability of 10.4%. Finally, when assessing ADC variability in normal healthy volunteers, no difference was observed between the right (9.7%) and left (9.8%) parotid glands used as a reference normal tissue in the head and neck areas.

The diagnostic accuracy of SUV_max_ parameter as an independent parameter for tumor prognosis is controversial owing to its reliability,^29^ therefore, it is reasonable to increase the SUV_max_ ‘s diagnostic accuracy using multimodal imaging technique, in particular, PET/MRI that can provide both SUV and ADC parameter at once. Especially, ADC parameter was extensively reported as a strong predictor of tumor prognosis.^30^, ^31^, ^32^

This study found that the majority of ADC and SUV variables of HNC acquired using simultaneous [^18^F]‐FDG PET/MRI had a negative and linear correlation; all SUV variables significantly increased with decrease in ADC_mean_, _med, 5, 10, 25,_ and _50_ values. SUV_max_ or SUV_peak_ had a strong correlation with ADC_mean, med,5,10,25,50_, variables and had a moderate correlation with ADC_min_, but not significantly correlated. The results of this study are similar to those of Brandmaier et al.,^33^ who reported the inverse correlation between SUV and ADC in cervical cancer (*r* = −0.532, *P* = 0.05). However, our study did not find a significant correlation between all statistic variables of SUV parameter and ADC_min_, ADC_max_, ADC_90,_ ADC_95,_ ADC_kur_, and ADC_skew_. This may be owing to an artifact or nontumor voxel, which normally lies in both histogram tails.

To compare extracted statistic variables of ADC with standard FDG‐based TLG and MTV parameters, we found that MTV and TLG tended to increase with the decrease of ADC values, except for ADC_max_. However, no statistical significance was observed owing to small number of sample size. ADC_max_ increased with an increased MTV, probably resulting from tissue necrosis within a tumor region.

Only a few published studies have reported the ADC and SUV of HNC correlation obtained by simultaneous PET/MRI technique for comparison. Varoquaux et al. (2013) found that the SUV increased with the decrease in ADC but not significantly correlated.^34^ They stated that ADC and SUV could be used as independent biomarkers for prognosis in head and neck squamous cell cancinoma without clinical outcome assessment. However, further investigation is still required from their conclusion, as unreliability of using SUV alone remains in current practice. To compare with this current study, the same trend of these two parameters was also found; however, we met a statistically significant correlation between most statistic variables of ADC and SUV. The improved results of this study may have been caused by simultaneously obtained images, which led to reduced confounding factors between two different scanners and time interval of scanning compared with their study, in which images were obtained from different scanners (PET/CT and MRI) and days of scanning.

Furthermore, the results from this current study could improve the limitation of using quantitative SUV alone in routine clinical practice for tumor interpretation. Injection time, serum blood glucose, volume averaging from motion, attenuation correction, and difference in scanners can vary SUV_max_ value, leading to erroneous interpretation of the results, especially, when SUV_max_ is used for tumor response. The significant correlation between ADC and SUV in simultaneous PET/MRI could allow to develop multimodal predictive model of tumor response.

This study has several limitations. First, we investigated a limited number of patients with HNC who underwent simultaneous PET/MRI. Increasing the sample size was suggested to improve the significance for further studies. Second, this study measured the ADC and SUV values of patients with heterogenous HNC, which may affect the alteration of the results. ADC and SUV measurement in a single type of HNC is suggested to improve the reliability of ADC and SUV parameters. Third, collected medical image data with radiological reports lacks the TNM classification of malignant tumors (TNM) information. As a result, we do not have this information for analysis in this current study. Lastly, we performed only 2D distortion correction provided by MRI vendor, median noise filtering, and image bias field correction without the correction of Gibbs ringing artefact, motion artifact, and eddy‐current problem that might alter ADC values in this current study. However, we excluded DWI‐MRI image data that suffered from patient motion from our data collection. We found that ADC variability was acceptable, although some diffusion image correction process was not carried out. The separated correction of Gibbs ringing artefact, motion correction, and eddy‐current correction would improve the reproducibility of ADC value in simultaneous PET/MRI in future study.

## 5 CONCLUSION

This study showed the feasibility of utilizing the SUV and ADC parameters derived from simultaneous PET/MRI as a quantitative PET/MRI parameter for assessing heterogeneity in HNC. The SUV and ADC parameters were reproducible, and their significant correlation would allow developing multimodal prediction model of tumor response in further clinical studies.

## AUTHOR CONTRIBUTION

PW (research project leader) and MP contributed to the conception and design of the work, management, analysis, data interpretation, revising the work critically for important intellectual content, agreement to be accountable for all aspects of the work, final approval of the version to be published, and manuscript preparing. MN, SS, and PP contributed equally to data collection, diffusion phantom development, image acquisition, image post‐processing, document preparation for ethic submission, healthy volunteer recruitment, and data analysis. AJ contributed to image data acquisition, data collection, and helping in manuscript preparing. DS contributed to patients’ data collection, tumor contouring, and helping in manuscript preparing. PL contributed to diffusion phantom development and helping in manuscript preparing (Supporting Information).

## CONFLICT OF INTEREST

The authors have no conflicts of interest to declare.

## Supporting information



Supporting Material

## References

[1] Bray F , Ferlay J , Soerjomataram I , Siegel RL , Torre LA . Global cancer statistics 2018: gLOBOCAN estimates of incidence and mortality worldwide for 36 cancers in 185 countries. CA Cancer J Clin. 2018;68(6):394‐424. 30207593 10.3322/caac.21492

[2] Aragón N , Ordoñez D , Urrea MF , Holguín J , Collazos P , García LS , et al. Head and neck cancer in Cali, Colombia: population‐based study. Community Dent Oral Epidemiol. 2022;50(4):292‐299. 34105170 10.1111/cdoe.12671 PMC8651828

[3] Siegel RLMK , Fuchs HE , Jemal A . Cancer statistics. CA Cancer J Clin. 2021;71(1):7‐33. 33433946 10.3322/caac.21654

[4] Cooney TR , MG Poulsen . Is routine follow‐up useful after combined‐ modality therapy for advanced head and neck cancer. Arch Otolaryngol Head Neck Surg. 1999;125(4):379‐382. 10208674 10.1001/archotol.125.4.379

[5] Hemprich A , RP Müller . Long‐term results in treating squamous cell carcinoma of the lip, oral cavity, and oropharynx. Int J Oral Maxillofac Surg. 1989;18(1):39‐42. 2497209 10.1016/s0901-5027(89)80014-1

[6] Nissi L , Suilamo S , Kytö E , et al. Recurrence of head and neck squamous cell carcinoma in relation to high‐risk treatment volume. Clin Transl Radiat Oncol. 2021;27:139‐146. 33665383 10.1016/j.ctro.2021.01.013 PMC7902285

[7] Eckardt A , Barth E , Kokemueller H , GJ Wegener . Recurrent carcinoma of the head and neck: treatment strategies and survival analysis in a 20‐year period. Oral Oncol. 2004;40(4):427‐432. 14969822 10.1016/j.oraloncology.2003.09.019

[8] Chicklore S , Goh V , Siddique M , et al. Quantifying tumour heterogeneity in 18F‐FDG PET/CT imaging by texture analysis. Eur J Nucl Med Mol Imaging. 2013;40(1):133‐140. 23064544 10.1007/s00259-012-2247-0

[9] Hinohara K , KJ Polyak . Intratumoral heterogeneity: more than just mutations. Trends Cell Biol. 2019;29(7):569‐579. 30987806 10.1016/j.tcb.2019.03.003 PMC6579620

[10] Chawla S , Kim S , Wang S , HJ Poptani . Diffusion‐weighted imaging in head and neck cancers. Future Oncol. 2009;5(7):959‐975. 19792966 10.2217/fon.09.77 PMC2791671

[11] van der Hoorn A , van Laar PJ , Holtman GA , Westerlaan HEJ . Diagnostic accuracy of magnetic resonance imaging techniques for treatment response evaluation in patients with head and neck tumors, a systematic review and meta‐analysis. PLoS One. 2017;12(5):e0177986. 28542474 10.1371/journal.pone.0177986 PMC5443521

[12] Surov A , Meyer HJ , Wienke AJO . Correlation between apparent diffusion coefficient (ADC) and cellularity is different in several tumors: a meta‐analysis. Oncotarget. 2017;8(35):59492. 28938652 10.18632/oncotarget.17752 PMC5601748

[13] Wang J , Takashima S , Takayama F , et al. Head and neck lesions: characterization with diffusion‐weighted echo‐planar MR imaging. Radiology. 2001;220(3):621‐630. 11526259 10.1148/radiol.2202010063

[14] Srinivasan A , Dvorak R , Perni K , Rohrer S , Mukherji SJ . Differentiation of benign and malignant pathology in the head and neck using 3T apparent diffusion coefficient values: early experience. AJNR Am J Neuroradiol. 2008;29(1):40‐44. 17921228 10.3174/ajnr.A0743 PMC8119114

[15] Maeda M , Kato H , Sakuma H , Maier SE , KJ Takeda . Usefulness of the apparent diffusion coefficient in line scan diffusion‐weighted imaging for distinguishing between squamous cell carcinomas and malignant lymphomas of the head and neck. AJNR Am J Neuroradiol. 2005;26(5):1186‐1192. 15891182 PMC8158607

[16] Meyer H‐J , Purz S , Sabri O , Surov AJ . Relationships between histogram analysis of ADC values and complex 18F‐FDG‐PET parameters in head and neck squamous cell carcinoma. PLoS One. 2018;13(9):e0202897. 30188926 10.1371/journal.pone.0202897 PMC6126801

[17] de Geus‐Oei LF , van der Heijden HF , Corstens FH , Oyen WJJC . Predictive and prognostic value of FDG‐PET in nonsmall‐cell lung cancer: a systematic review. Cancer. 2007;110(8):1654‐1664. 17879371 10.1002/cncr.22979

[18] Dehdashti F , Mortimer JE , Trinkaus K , et al. PET‐based estradiol challenge as a predictive biomarker of response to endocrine therapy in women with estrogen‐receptor‐positive breast cancer. Breast Cancer Res Treat. 2009;113(3):509‐517. 18327670 10.1007/s10549-008-9953-0 PMC3883567

[19] Mac Manus MP , Ding Z , Hogg A , et al. Association between pulmonary uptake of fluorodeoxyglucose detected by positron emission tomography scanning after radiation therapy for non–small‐cell lung cancer and radiation pneumonitis. J Radiat Oncol Biol Phys. 2011;80(5):1365‐1371. 10.1016/j.ijrobp.2010.04.021 20675076

[20] Schmeel FCJ . Variability in quantitative diffusion‐weighted MR imaging (DWI) across different scanners and imaging sites: is there a potential consensus that can help reducing the limits of expected bias? Eur Radiol. 2019:2243‐2245. 30488105 10.1007/s00330-018-5866-4

[21] Koopman D , Jager PL , Slump CH , Knollema S , van Dalen JAJ . SUV variability in EARL‐ accredited conventional and digital PET. EJNMMI Res. 2019;9(1):1‐8. 31823097 10.1186/s13550-019-0569-7 PMC6904705

[22] Fahey FH , Kinahan PE , Doot RK , Kocak M , Thurston H , Poussaint TYJ . Variability in PET quantitation within a multicenter consortium. Med Phys. 2010;37(7Part1):3660‐3666. 20831073 10.1118/1.3455705 PMC2905446

[23] Schlett CL , Hendel T , Hirsch J , Weckbach S , Caspers S , Schulz‐Menger J , et al. Quantitative, organ‐specific interscanner and intrascanner variability for 3 T whole‐body magnetic resonance imaging in a multicenter. Multivendor Study. 2016;51(4):255‐265. 10.1097/RLI.0000000000000237 26646309

[24] Fahey FH , Kinahan PE , Doot RK , Kocak M , Thurston H , Poussaint TY . Variability in PET quantitation within a multicenter consortium. Med Phys. 2010;37(7Part1):3660‐3666. 20831073 10.1118/1.3455705 PMC2905446

[25] Koopman D , Jager PL , Slump CH , Knollema S , van Dalen JA . SUV variability in EARL‐ accredited conventional and digital PET. EJNMMI Research. 2019;9(1):106. 31823097 10.1186/s13550-019-0569-7 PMC6904705

[26] Hakulinen U , Brander A , Ryymin P , et al. Repeatability and variation of region‐of‐interest methods using quantitative diffusion tensor MR imaging of the brain. BMC Medical Imaging. 2012;12(1):30. 23057584 10.1186/1471-2342-12-30 PMC3533516

[27] Kanstrup IL , Klausen TL , Bojsen‐Møller J , Magnusson P , Zerahn B . Variability and reproducibility of hepatic FDG uptake measured as SUV as well as tissue‐to‐blood background ratio using positron emission tomography in healthy humans. Clinical physiology and functional imaging. 2009;29(2):108‐113. 19076727 10.1111/j.1475-097X.2008.00846.x

[28] Hara M , Kuroda M , Ohmura Y , et al. A new phantom and empirical formula for apparent diffusion coefficient measurement by a 3 Tesla magnetic resonance imaging scanner. Oncol Lett. 2014;8(2):819‐824. 25013504 10.3892/ol.2014.2187 PMC4081373

[29] Kitajima K , Suenaga Y , Minamikawa T , et al. Clinical significance of SUVmax in (18)F‐FDG PET/CT scan for detecting nodal metastases in patients with oral squamous cell carcinoma. SpringerPlus. 2015;4:718. 26636006 10.1186/s40064-015-1521-6 PMC4656255

[30] Mohamed A , Abusaif A , He R , et al. Prospective Assessment of diffusion‐weighted‐MRI as a biomarker of treatment response and disease control in head and neck cancer. Neurology. 2022;114(3):S49.

[31] Wang Y , Yu T , Yang Z , et al. Radiomics based on magnetic resonance imaging for preoperative prediction of lymph node metastasis in head and neck cancer: machine learning study. Eur J Med Res. 2022;44(12):2786‐2795. 10.1002/hed.27189 36114765

[32] Kim M , Lee JH , Joo L , et al. Development and validation of a model using radiomics features from an apparent diffusion coefficient map to diagnose local tumor recurrence in patients treated for head and neck squamous cell carcinoma. Korean J Radiol. 2022;23(11):1078‐1088. 36126954 10.3348/kjr.2022.0299 PMC9614290

[33] Brandmaier P , Purz S , Bremicker K , et al. Simultaneous [18F] FDG‐PET/MRI: correlation of apparent diffusion coefficient (ADC) and standardized uptake value (SUV) in primary and recurrent cervical cancer. PLoS One. 2015;10(11):e0141684. 26551527 10.1371/journal.pone.0141684 PMC4638340

[34] Varoquaux A , Rager O , Lovblad K‐O , et al. Functional imaging of head and neck squamous cell carcinoma with diffusion‐weighted MRI and FDG PET/CT: quantitative analysis of ADC and SUV. Eur J Nucl Med Mol Imaging. 2013;40(6):842‐852. 23436068 10.1007/s00259-013-2351-9 PMC3644194

