# Supplementary material for: Assessing the variability and correlation between SUV and ADC parameters of head and neck cancers derived from simultaneous PET/MRI: A single‐center study
Source: J Appl Clin Med Phys. 2023 Feb 10;24(5):e13928. doi: 10.1002/acm2.13928 (PMC10161023; doi:10.1002/acm2.13928)
Supplement: Supplementary file 1 — Supporting Material [file ACM2-24-e13928-s001.docx]

**Supplementary Tables**

| **Table s1.** Average SUV value measured from NEMA Phantom and inter-session CV value of each sphere | | | | |
| --- | --- | --- | --- | --- |
| spheres | mean SUV | | | inter–session CV (%) |
|  | session 1 | session 2 | session 3 |  |
| 1 | 3.24 | 3.40 | 3.03 | 5.8 |
| 2 | 4.33 | 5.05 | 4.38 | 8.7 |
| 3 | 5.29 | 6.97 | 5.97 | 13.9 |
| 4 | 8.88 | 9.43 | 8.47 | 5.5 |
| 5 | 2.70 | 2.84 | 2.35 | 9.7 |
| 6 | 2.91 | 3.37 | 2.91 | 8.6 |
| **Abbreviation:** * SUV, Standardized Uptake Value; CV, Coefficient of Variation | | | | |

| **Table s2.** Average ADC measured from inhouse diffusion phantom and intrasession CV of session1 and intersession CV | | | | | | | | | |
| --- | --- | --- | --- | --- | --- | --- | --- | --- | --- |
| Sessions | Acquisitions | mean ADC (×10^-3^ mm^2^/s) of each sucrose concentration | | | | | | | ADC of gel |
|  |  | 0 | 0.2 | 0.4 | 0.6 | 0.8 | 1 | 1.2 |  |
| 1 | 1 | 2.70 | 2.54 | 2.58 | 2.49 | 1.60 | 1.67 | 1.55 | 2.62 |
|  | 2 | 2.50 | 2.41 | 2.51 | 2.36 | 1.55 | 1.63 | 1.44 | 2.49 |
|  | 3 | 2.22 | 2.09 | 2.23 | 2.11 | 1.56 | 1.55 | 1.28 | 2.16 |
| 2 | 1 | 2.22 | 2.18 | 2.31 | 2.19 | 1.77 | 1.81 | 1.48 | 2.24 |
|  | 2 | 2.41 | 2.30 | 2.43 | 2.33 | 1.61 | 1.59 | 1.42 | 2.33 |
|  | 3 | 2.88 | 2.78 | 2.81 | 2.77 | 1.77 | 1.71 | 1.65 | 2.87 |
| 3 | 1 | 2.57 | 2.43 | 2.54 | 2.47 | 1.93 | 1.78 | 1.50 | 2.43 |
|  | 2 | 2.27 | 2.12 | 2.28 | 2.21 | 2.15 | 1.82 | 1.50 | 2.22 |
|  | 3 | 2.58 | 2.48 | 2.64 | 2.47 | 1.78 | 1.85 | 1.59 | 2.49 |
| Intrasession CV of Session1 (%) | | 9.7 | 9.8 | 7.5 | 8.4 | 1.9 | 3.7 | 9.7 | 9.8 |
| Intersession CV (%) | | 10.0 | 10.4 | 8.4 | 9.0 | 5.6 | 4.0 | 7.0 | 9.8 |

| **Table s3.** Average ADC measured from both Parotid glands | | | | | |
| --- | --- | --- | --- | --- | --- |
| average ADC (×10^-3^ mm^2^/s) | | | | | |
| volunteers | Rt. Parotid gland | |  | Lt. Parotid gland | |
|  | session 1 | session 2 |  | session 1 | session 2 |
| 1 | 1.20 | 1.15 |  | 1.17 | 1.19 |
| 2 | 1.37 | 1.40 |  | 1.36 | 1.39 |
| 3 | 1.21 | 1.19 |  | 1.19 | 1.12 |
| 4 | 1.14 | 1.21 |  | 1.21 | 1.24 |
| 5 | 1.07 | 1.03 |  | 1.09 | 1.01 |
| Inter–subject (%) | 9.7 | |  | 9.8 | |

| **Table s4**. P-value of the correlation between ADC and SUV variables | | | | | | | | | | | | | |
| --- | --- | --- | --- | --- | --- | --- | --- | --- | --- | --- | --- | --- | --- |
| **variable** | **ADC_mean_** | **ADC_med_** | **ADC_min_** | **ADC_max_** | **ADC_5_** | **ADC_10_** | **ADC_25_** | **ADC_50_** | **ADC_75_** | **ADC_90_** | **ADC_95_** | **ADC_ske_** | **ADC_kur_** |
| **MTV** | 0.35 | 0.20 | 0.05 | 0.07 | 0.10 | 0.12 | 0.12 | 0.20 | 0.63 | 0.77 | 0.57 | 0.86 | 0.72 |
| **TLG** | 0.24 | 0.12 | 0.10 | 0.16 | 0.07 | 0.08 | 0.07 | 0.12 | 0.48 | 0.95 | 0.74 | 0.73 | 0.71 |
| **SUV_mean_** | 0.02* | 0.01* | 0.43 | 0.67 | 0.02* | 0.01* | 0.01* | 0.01* | 0.05* | 0.09 | 0.19 | 0.51 | 0.70 |
| **SUV_med_** | 0.02* | 0.01* | 0.37 | 0.75 | 0.02* | 0.01* | 0.01* | 0.01* | 0.05* | 0.11 | 0.21 | 0.52 | 0.77 |
| **SUV_min_** | 0.25 | 0.29 | 0.54 | 0.10 | 0.48 | 0.38 | 0.41 | 0.29 | 0.20 | 0.08 | 0.10 | 0.73 | 0.61 |
| **SUV_max_** | 0.03* | 0.01* | 0.22 | 0.86 | 0.01* | 0.01* | 0.01* | 0.01* | 0.08 | 0.20 | 0.37 | 0.50 | 0.78 |
| **SUV_5_** | 0.11 | 0.11 | 0.97 | 0.23 | 0.19 | 0.14 | 0.16 | 0.11 | 0.11 | 0.06 | 0.10 | 0.72 | 0.61 |
| **SUV_10_** | 0.09 | 0.08 | 0.87 | 0.31 | 0.15 | 0.11 | 0.12 | 0.08 | 0.09 | 0.07 | 0.12 | 0.63 | 0.57 |
| **SUV_25_** | 0.03* | 0.02* | 0.58 | 0.45 | 0.05* | 0.03* | 0.03* | 0.02* | 0.05* | 0.06 | 0.13 | 0.56 | 0.61 |
| **SUV_50_** | 0.02* | 0.01* | 0.37 | 0.75 | 0.02* | 0.01* | 0.01* | 0.01* | 0.05* | 0.11 | 0.21 | 0.52 | 0.77 |
| **SUV_75_** | 0.02* | 0.01* | 0.34 | 0.84 | 0.02* | 0.01* | 0.01* | 0.01* | 0.06 | 0.13 | 0.25 | 0.54 | 0.83 |
| **SUV_90_** | 0.03* | 0.01* | 0.36 | 0.84 | 0.02* | 0.01* | 0.01* | 0.01* | 0.06 | 0.13 | 0.26 | 0.48 | 0.72 |
| **SUV_95_** | 0.03* | 0.01* | 0.33 | 0.88 | 0.02* | 0.01* | 0.01* | 0.01* | 0.06 | 0.14 | 0.27 | 0.47 | 0.70 |
| **SUV_ske_** | 0.42 | 0.33 | 0.37 | 0.81 | 0.40 | 0.36 | 0.27 | 0.33 | 0.56 | 0.84 | 1.00 | 0.83 | 0.88 |
| **SUV_kur_** | 0.44 | 0.46 | 0.97 | 0.33 | 0.59 | 0.51 | 0.46 | 0.46 | 0.49 | 0.37 | 0.35 | 0.86 | 0.86 |
| * statistically significant correlation at p-value of <0.05 using Pearson’s correlation | | | | | | | | | | | | | |

**Table s5.** The 95% confidence interval of r-value across the ADC and SUV variables

| **variable** | **ADC_mean_** | **ADC_med_** | **ADC_min_** | **ADC_max_** | **ADC5** | **ADC10** | **ADC25** | **ADC50** | **ADC75** | **ADC90** | **ADC95** | **ADC_ske_** | **ADC_kur_** |
| --- | --- | --- | --- | --- | --- | --- | --- | --- | --- | --- | --- | --- | --- |
| **MTV** | -0.77, 0.36 | -0.81,0.24 | -0.88,0.00 | -0.05,0.87 | -0.85,0.11 | -0.84,0.15 | -0.85,0.14 | -0.81,0.24 | -0.70,0.48 | -0.53,0.66 | -0.46,0.71 | -0.56,0.64 | -0.67,0.52 |
| **TLG** | -0.80,0.13 | -0.85, -0.25 | -0.85,0.40 | -0.20,0.50 | -0.87,0.50 | -0.86, -0.12 | -0.87,0.05 | -0.85,0.15 | -0.73,0.42 | -0.59,0.61 | -0.52,0.67 | -0.52,0.67 | -0.67,0.51 |
| **SUV_mean_** | -0.91, -0.13 | -0.93,0.25 | -0.751, -0.40 | -0.69-0.50 | -0.91, -0.12 | -0.92,0.20 | -0.92,0.21 | -0.93, -0.25 | -0.88,0.01 | -0.86, 0.10 | -0.82, 0.23 | -0.44, 0.73 | -0.51, -0.68 |
| **SUV_med_** | -0.91, -0.14 | -0.93, -0.29 | -0.76, -0.37 | -0.67, -0.52 | -0.91, -0.17 | -0.93, -0.24 | -0.93, -0.26 | -0.93, -0.29 | -0.88, -0.01 | -0.85, -0.13 | -0.81, -0.25 | -0.44, -0.72 | -0.53, -0.66 |
| **SUV_min_** | -0.80, -0.29 | -0.79, -0.31 | -0.45,0.72 | -0.86,0.11 | -0.73,0.42 | -0.76,0.37 | -0.75,0.39 | -0.79,0.31 | -0.82,0.24 | -0.87,0.07 | -0.86,0.11 | -0.52,0.67 | -0.48,0.70 |
| **SUV_max_** | -0.90, 0.09 | -0.93, -0.27 | -0.81, 0.26 | -0.56, 0.64 | -0.92, -0.20) | -0.93, -0.25) | -0.93, -0.28) | -0.93, -0.27) | -0.87, -0.07 | -0.81, -0.25 | -0.76, -0.37 | -0.43, -0.73 | -0.53, -0.66 |
| **SUV5** | -0.85, -0.13 | -0.85, -0.13 | -0.61, -0.59 | -0.81, -0.27 | -0.82, -0.24 | -0.83, -0.18 | -0.83, -0.20 | -0.85, -0.13 | -0.85, -0.12 | -0.88, -0.03 | -0.85, -0.11 | -0.51, -0.67 | -0.48, -0.70 |
| **SUV10** | -0.86, -0.10 | -0.87, -0.07 | -0.63, -0.56 | -0.78, -0.33 | -0.83, -0.19 | -0.85, -0.13 | -0.85, -0.14 | -0.87, -0.07 | -0.86, -0.10 | -0.87, -0.05 | -0.84, -0.15 | -0.48, -0.70 | -0.46, -0.71 |
| **SUV25** | -0.90, -0.08 | -0.91, -0.16 | -0.71, -0.46 | -0.74, -0.41 | -0.89, -0.02 | -0.90, -0.09 | -0.90, -0.09) | -0.91, -0.16 | -0.89, -0.02 | -0.88, -0.02 | -0.84, -0.15 | -0.46, -0.71 | -0.47, -0.70 |
| **SUV50** | -0.91, -0.14 | -0.93, -0.29 | -0.76, -0.37 | -0.67, -0.52 | -0.91, -0.17 | -0.93, -0.24 | -0.93, -0.26 | -0.93, -0.29 | -0.88, -0.01 | -0.85, -0.13 | -0.81-0.25 | -0.44, -0.72 | -0.53, -0.66 |
| **SUV75** | -0.91, -0.12 | -0.93, -0.28 | -0.77, -0.35 | -0.64, -0.55 | -0.92, -0.18 | -0.93, -0.24 | -0.93, -0.26 | -0.93, -0.28 | -0.88, -0.03 | -0.84, -0.17 | -0.80, -0.29 | -0.45, -0.72 | -0.55, -0.64 |
| **SUV90** | -0.90, -0.11 | -0.93, -0.26 | -0.77, -0.36 | -0.64, -0.56 | -0.91, -0.14 | -0.92, -0.21 | -0.93, -0.24 | -0.93, -0.26) | -0.88, -0.03 | -0.84, -0.17 | -0.80, -0.29 | -0.42, -0.73 | -0.52, -0.67 |
| **SUV95** | -0.90, -0.10 | -0.93, -0.26 | -0.77, -0.34 | -0.63, -0.56 | -0.91, -0.14 | -0.92, -0.21 | -0.93, -0.24 | -0.93, -0.26) | -0.88, -0.03 | -0.84, -0.17 | -0.79, -0.30 | -0.42, -0.74 | -0.51, -0.68 |
| **SUV_ske_** | -0.39, -0.75 | -0.34, -0.77 | -0.36, -0.76 | -0.65, -0.54 | -0.38, -0.75 | -0.36, -0.77 | -0.30, -0.79 | -0.34, -0.77 | -0.45, -0.71 | -0.55, -0.64 | -0.60, -0.60 | -0.64, -0.55 | -0.57, -0.63 |
| **SUV_kur_** | -0.40, -0.74 | -0.41, -0.74 | -0.59, -0.61 | -0.34, -0.77 | -0.47, -0.71 | -0.43, -0.73 | -0.41, -0.74 | -0.41, -0.74 | -0.42, -0.73 | -0.36, -0.76 | -0.36, -0.77 | -0.56, -0.64 | -0.56, -0.64 |
